# Resource Allocation for Maximizing Prediction Accuracy and Genetic Gain of Genomic Selection in Plant Breeding: A Simulation Experiment

**DOI:** 10.1534/g3.112.004911

**Published:** 2013-03-01

**Authors:** Aaron J. Lorenz

**Affiliations:** Department of Agronomy and Horticulture, University of Nebraska, Lincoln, Nebraska 68583

**Keywords:** genomic selection, plant breeding, GenPred, Shared data resources

## Abstract

Allocating resources between population size and replication affects both genetic gain through phenotypic selection and quantitative trait loci detection power and effect estimation accuracy for marker-assisted selection (MAS). It is well known that because alleles are replicated across individuals in quantitative trait loci mapping and MAS, more resources should be allocated to increasing population size compared with phenotypic selection. Genomic selection is a form of MAS using all marker information simultaneously to predict individual genetic values for complex traits and has widely been found superior to MAS. No studies have explicitly investigated how resource allocation decisions affect success of genomic selection. My objective was to study the effect of resource allocation on response to MAS and genomic selection in a single biparental population of doubled haploid lines by using computer simulation. Simulation results were compared with previously derived formulas for the calculation of prediction accuracy under different levels of heritability and population size. Response of prediction accuracy to resource allocation strategies differed between genomic selection models (ridge regression best linear unbiased prediction [RR-BLUP], BayesC*π*) and multiple linear regression using ordinary least-squares estimation (OLS), leading to different optimal resource allocation choices between OLS and RR-BLUP. For OLS, it was always advantageous to maximize population size at the expense of replication, but a high degree of flexibility was observed for RR-BLUP. Prediction accuracy of doubled haploid lines included in the training set was much greater than of those excluded from the training set, so there was little benefit to phenotyping only a subset of the lines genotyped. Finally, observed prediction accuracies in the simulation compared well to calculated prediction accuracies, indicating these theoretical formulas are useful for making resource allocation decisions.

A critical aspect to the design of plant breeding programs is the allocation of limited resources between population size and replication. Larger population sizes allow greater selection intensity and probability of identifying superior recombinants, whereas increased replication improves heritability, especially for highly complex traits prone to measurement error and random environmental deviations. For such traits, such as grain yield, theory suggests at least some replication across relevant environments should be conducted, the exact amount depending on the resource level and heritability of single plot measurements (Wricke and Weber 1986; Moreau *et al.* 2000). Level of replication also depends upon stage of the breeding pipeline. It is common practice to evaluate progenies using less replication during early testing and save more resources for greater replication of fewer lines during later testing when parent and cultivar release decisions are to be made (Bernardo 2010). The bottom line is that selection decisions made upon phenotypic information solely require extensive replication across the target population of environments to ensure the genotypic values of the selection candidates have been precisely estimated.

When the purpose of phenotypic evaluations turn from informing phenotypic selection to calibrating statistical models for genomic selection, the unit of evaluation switches from the whole genotypic value of a progeny to the additive genetic value of a single allele. Hence, so-called hidden replication of alleles can be increased by increasing population size. It is well known that power and precision of quantitative trait loci (QTL) mapping and marker effect estimation is greatly influenced by population size and heritability (Yu *et al.* 2008; Beavis 1994; Gimelfarb and Lande 1994; Schon *et al.* 2004). Surprisingly few studies have thoroughly studied the tradeoff in detection power and effect estimation between increasing population size and increasing heritability through additional replication. Knapp and Bridges (1990) analyzed the expected mean squares of a model describing the sources of variation included in a QTL mapping experiment with replicated progenies and calculated power of QTL detection for different resource allocation strategies, not considering the cost of genotyping. They showed that when residual genetic variation exists within groups of progeny sharing QTL genotypes, power is most efficiently maximized by increasing population size at the expense of replication. Using real data from a very large QTL mapping experiment, Schon *et al.* (2004) found that increasing population size generally increased QTL detection power more than increasing number of test environments. For more typical QTL mapping population sizes (*i.e.*, 150−300), some degree of replication is needed for some QTL detection power (Schon *et al.* 2004).

Genomic selection is a marker-based selection method that uses all available marker information to predict genetic value of breeding progenies for selection. By using winter nursery and greenhouse facilities, it is possible to make at least two to three generations of selection and recombination per year in annual crop species. In temperate environments, only one relevant phenotypic measurement can be made per year, meaning that marker effect estimates applied to progeny selections need to be robust across generations of recombination to maximize genetic gain per unit time. Many studies on genomic selection in plants have been performed using deterministic calculations (Heffner *et al.* 2010), simulations (Bernardo and Yu 2007; Zhong *et al.* 2009; Jannink 2010), and empirical results (Lorenzana and Bernardo 2009; Heffner *et al*, 2011; Lorenz *et al.* 2012), but none have explicitly investigated the effect of experimental resource allocation choices during model development on total genetic gain and accuracy of genomic selection models for predicting genetic value of progenies at least one generation of recombination removed from the training population. Some work on resource allocation has been performed on traditional marker-assisted selection (Moreau *et al.* 2000). In line with the aforementioned QTL detection power results, these authors showed that when genotyping and phenotyping costs are approximately equal, maximum gain from marker-assisted selection is achieved when more resources are allocated to larger population sizes rather than replication. However, some key differences in assumptions could preclude the findings of Moreau *et al.* (2000) from being generalized to genomic selection. First, these authors consider a trait being controlled by only 5−10 QTL. Genomic selection is aimed at and works best for traits being controlled by many more QTL [*e.g.*, 100 (Bernardo and Yu 2007)]. Second, Moreau *et al.* (2000) consider marker effects as fixed effects and used a statistical threshold for deciding which markers to include in the marker score. Thus, proportion of genetic variance described by markers is closely related to power of QTL detection. Genomic selection, on the other hand, does not apply a statistical threshold for choosing markers, but rather estimates effects of all markers simultaneously and sums across all markers to obtain the marker score.

This paper reports the results of simulations aimed at exploring the effect of resource allocation choices, in terms of population size and replication, on (1) prediction accuracy and (2) total genetic gain of genomic selection models applied to progenies in a genomic recurrent selection scheme. Results from simulations are also compared to predictions made using available theoretical formulas.

## Materials and Methods

### Theory

According to basic selection theory, response to selection is equal to the product of the selection intensity (*i*), selection accuracy (*r*_A_), and SD of breeding values ($\sigma_{A}$).

$$R=ir_{A}\sigma_{A}$$

The selection accuracy is defined as the correlation between true breeding value and estimated breeding value (EBV). When genome-wide marker information is used to construct a prediction, the EBV is referred to as the genomic EBV (GEBV). The accuracy of GEBVs is the most important criterion for evaluating the potential usefulness of genomic selection and for comparing different GS models and strategies. Equations for the calculation of GS accuracy before data collection have been derived by Goddard (2009) and Daetwyler *et al.* (2008). Extensions and alternative formulations have been provided by Goddard *et al.* (2011) and Hayes *et al.* (2009).

Consider that the true breeding value of individual *i* is $a_{i}=\sum z_{ij}\alpha_{j}$ where $z_{ij}$ is the allelic state at locus j and $z_{ij}\varepsilon \{-1,0,1\}$, $\alpha_{j}$ is the true allelic substitution effect at locus j. Similarly, ${\hat{a}}_{i}=\sum_{j}z_{ij}{\hat{\alpha }}_{j}$ represents the estimated breeding value, where ${\hat{\alpha }}_{j}$ is the estimated substitution effect at locus j obtained by regressing the phenotypes on the allelic state at locus j. The goal is to express

(1) $$r_{A}=\frac{\mathrm{cov}\left(a_{i},{\hat{a}}_{i}\right)}{\sqrt{\mathrm{var}(a_{i})\mathrm{var}({\hat{a}}_{i})}}$$

in terms of population size and trait heritability so that effects of resource allocation on genomic selection accuracy can be calculated before data collection.

Using basic principles in quantitative genetics and simple linear regression and making a couple of conservative assumptions, Daetwyler *et al.* (2008) showed that

(2) $$r_{a\hat{a}}=\sqrt{\frac{\frac{nh^{2}}{m_{e}}}{\frac{nh^{2}}{m_{e}}+1}},$$

where *n* is the number of individuals with phenotypes in the training population, $m_{e}$ is the effective number of factors (or loci) for which substitution effects need to be estimated, and *h*^2^ is the trait heritability. Some discussion on $m_{e}$ is provided herein. It is assumed the individuals used to estimate allele substitution effects and the individuals whose genetic value is being predicted are different sets of individuals but are from the same population. Also, this formula is derived by considering that the effect of each locus is estimated by regressing phenotypes on genotypes one locus at a time. The error variance is reduced by fitting all loci simultaneously. Daetwyler *et al.* (2008) proposed an approximate adjustment of $0.5r_{a\hat{a}}^{4}$ (*m_e_/n*), so that

(3) $$r_{a\hat{a}}\approx \frac{1}{2}{\left(\frac{\frac{nh^{2}}{m_{e}}}{\frac{nh^{2}}{m_{e}}+1}\right)}^{2}\frac{m_{e}}{n}$$

Goddard and co-workers (Goddard 2009; Hayes *et al.* 2009; Goddard *et al.* 2011) derived the same formula but took a slightly different approach based on the genomic relationship between individuals in the training population and selection candidates, which is used in place of a pedigree-based additive relationship matrix in the standard animal model.

### Effective number of loci

Effective number of loci is defined and used to derive genomic prediction accuracy formulas. This quantity represents how many essentially independent effects need to be estimated. It is a function of the rate of linkage disequilibrium decay in a population, and is equivalent to the number of freely segregating loci of equal effect giving rise to the observed population range and genetic variance (Lynch and Walsh 1998). The effective number of loci cannot be greater than the number of independently segregating chromosomal segments. In complex, randomly mating populations, the number of independently segregating chromosomal segments is a function of genome length and effective population size (Hayes *et al.* 2009). Within families, this quantity is related to the degree of relationship between family members, genome size, and chromosome number. In double haploid populations derived from an F1, the number of independently segregating chromosomal segments can be calculated as the haploid number of chromosome plus the expected number of cross-over events (Lynch and Walsh 1998). The expected number of cross-over events is equal to the genome length in Morgans.

Equation 3 was used to calculate expected prediction accuracy under various combinations of *n* and r. The population modeled was a population of DH lines from a biparental cross of inbred parents. The genome of maize consists of 10 chromosomes with a total genetic distance of 1796 cMs (Genetic 2008 composite map; www.maizegdb.org), resulting in 28 effective loci.

### Simulations

A breeding program applying genomic predictions to recurrent selection within a maize biparental population was simulated. This is essentially a marker-assisted recurrent selection strategy (Bernardo 2008), but molecular marker scores comprise summation of all marker effects rather than just those determined to be significant in a QTL analysis. The genetic distance of each chromosome was set according to the Genetic 2008 composite map found on the Maize Genetics and Genomics Database (www.maizegdb.org). The total genetic distance of the Genetic 2008 map is 1796 cM. Five-hundred single-nucleotide polymorphisms (SNPs) were simulated per chromosome. One-hundred random SNPs across the genome were designated as QTL. QTL additive effects were calculated according to a geometric series as in Bernardo and Yu (2007). All genetic variance was simulated as additive genetic variance. Two hundred evenly distributed SNPs were designated as markers. A preliminary analysis found increases in *r*_A_ with additional markers plateaued around 200 markers (data not shown).

A population of DH lines derived from the F1 between two parental lines polymorphic at all loci was simulated. Negative and positive values of QTL alleles were randomly assigned to parents. The genetic value of each DH line was calculated by summing effects of all QTL alleles carried by an individual. Measurements of single plots were simulated by adding a random environmental deviation to the genetic value. Environmental deviations were normally distributed with variance $\sigma_{e}^{2}=\sigma_{a}^{2}/h_{plot}^{2}-\sigma_{a}^{2}$, where $\sigma_{a}^{2}$ represents the additive genetic variance and $h_{plot}^{2}$ represents the heritability of single plot values. For marker effect estimation, the mean of *r* plot values per DH line was calculated.

Where experimental design parameters were constrained by a budget, the tradeoff between replication number and population size was made according to a budget expressed in units of field plots (B), B=n(C+rF), where C is the cost (in plots) of genotyping a DH line, *n* is the number of DH lines, *r* is the number of replications, and *F* is the proportion of DH lines placed in field trials. Levels of *F* considered were 1, 0.75, and 0.50.

Two uses of the DH population to investigate model accuracy were deployed: cross-validation and selection-recombination simulation. Cross-validation involved splitting the DH population into a model training set and validation set. The size of the model training set (*n*) was variable. Five-hundred DH lines comprised the validation set. Phenotypes in the training set were used for marker effect estimation. GEBVs of the validation set were calculated by summing estimated marker allele effects for each DH line. Prediction accuracy was calculated as the correlation between validation-set GEBVs and true genetic values.

Simulation of selection and recombination involved phenotyping a DH population of size N, training a genomic selection model on that phenotypic data, and performing at least three generations of selection and recombination. Although phenotypic data are available on Cycle 0 (C0) DH lines, genomic predictions were used for selection because preliminary analyses showed selection on markers alone was more effective in C0 compared with either phenotypic selection or selection on an index combining phenotypes and genomic predictions (data not shown). In the first cycle, 10 DH lines were selected and randomly crossed to form 5 random F1s. The F1s were randomly mated to create 200 C1 S0 plants. The genomic selection model trained using C0 DHs was applied to the 200 C1 progenies to select 20 C1 S0 plants with the highest GEBVs. Selected plants were recombined to form C2. This procedure was repeated to create C3. Total genetic gain was calculated as the difference between C0 and C3 average genetic value.

Because different levels of *n* inherent in this study cause differences in selection intensity and thus genetic variation in subsequent cycles, it is impossible to examine the influence of *n* and *r* on model accuracy in subsequent cycles. To circumvent this issue and isolate the effect of varying resource allocation on model prediction accuracy in subsequent cycles, another set of simulations was performed without the aspect of selection. The DH lines of C0 were randomly mated with replacement to create 1000 F1s, which were randomly mated to create 1000 C1 S0 plants. This procedure was continued to create C2 and C3 and ensured that allele frequencies were stable and that genetic variation present in the C0 population was held constant, allowing a comparison of different resource allocation options in producing a genomic selection model with predictive accuracy of populations more than one generation removed from the training population.

All simulations were performed using the statistical software package R. Genetic recombination was performed using functions obtained from the R package *hypred*. All routines were repeated at least 200 times, starting with simulation of population and genetic architecture. Genetic gain evaluations were performed with RR-BLUP only, which is computationally less intensive, and therefore routines for this part of the study were run 500 times. Prediction accuracies and genetic gains were averaged across iterations and standard errors of the means were calculated. An R script containing functions used in the simulation and a user script executing the functions are provided as Supporting Information, File S1 and File S2.

### Models

Because all the data in the simulations are balanced, and all individuals belong to the same biparental family, the basic model relating marker scores to phenotypes can be represented as $y_{i}=\mu +\sum_{m}z_{ij}u_{j}\delta_{j}+e_{i}$, where *y*_i_ represents the entry mean of the *i*^th^ DH line, *μ* is the population mean, *z*_ij_ is the allelic state of marker *j* for individual *i* coded as {A_1_A_1_, A_2_A_2_} = {−1, 1}, *u*_j_ is the estimated effect of marker *j*, $\delta_{j}$ is an indicator variable for the inclusion of marker *j*, and *e*_i_ is a residual.

Three models were used: ridge regression best linear unbiased prediction (RR-BLUP), BayesC*π* (Habier *et al.* 2011), and a multiple regression model in which marker effects were estimated with ordinary least squares (OLS). In RR-BLUP, $\delta_{j}$ = 1 and $u_{j}∼N(0,\sigma_{u}^{2})$, where $\sigma_{u}^{2}$ is estimated by maximum likelihood. This means that all marker effects are sampled from the same distribution and thus are shrunken toward 0 to the same degree. The R package *rrBLUP* developed by Endelman (2011) was used to implement the model. This implementation estimates $\sigma_{u}^{2}$ through the spectral decomposition algorithm of Kang *et al.* (2008). BayesC*π* is a modification of BayesB. Under BayesC*π*, $\delta_{j}$ = 0 with probability *π*, $\delta_{j}$ = 1 with probability 1 – *π* and $u_{j}∼N(0,\sigma_{u}^{2})$. Parameter *π* itself is estimated from the data. The prior distribution of *π* is uniform between 0 and 1. The method estimates $\sigma_{u}^{2}$ jointly over all nonzero markers (Kizilkaya *et al* 2010). The previous distribution for $\sigma_{u}^{2}$ follows a scaled inverse χ^2^ distribution with 4 df ($\nu_{u}$) and scale parameter $S_{u}^{2}=[(\nu_{u}-2){\tilde{\sigma }}_{u}^{2}]/\nu_{u}$, where ${\tilde{\sigma }}_{u}^{2}$ represents the variance of additive effects of a random sampled locus.

(4) $${\tilde{\sigma }}_{u}^{2}=\frac{\sigma_{\overset{–}{P}}^{2}}{(200\times (1-\pi ){\overset{–}{\sigma }}_{m}^{2}}$$

where $\sigma_{\overset{–}{P}}^{2},$ represents the variance of DH line means, 200 is equal to the number of markers, and ${\overset{–}{\sigma }}_{m}^{2}$ represents the marker score variance averaged across loci.

Three thousand Markov chain iterations were run, with 1000 discarded as burn-in. This was judged to be sufficient because previous results showed stable estimates and because all parameters of interest in the simulation were estimated by running all simulations at least 200 times. The OLS model was selected using a forward-backward model selection algorithm. All markers were first fit individually. The marker with the lowest *p*-value was added to the model, and each remaining marker was individually fitted again. The next marker with the lowest *p*-value was then added to the model and this process was repeated. After each round of forward selection, backward elimination was performed by eliminating any markers upon model refit with significance levels that dropped below the statistical threshold. The *p*-value for both forward selection and backward elimination was set to 0.20. This algorithm was iterated until convergence.

## Results

### Effect of replication and population size on prediction accuracy

**Figure 1:**
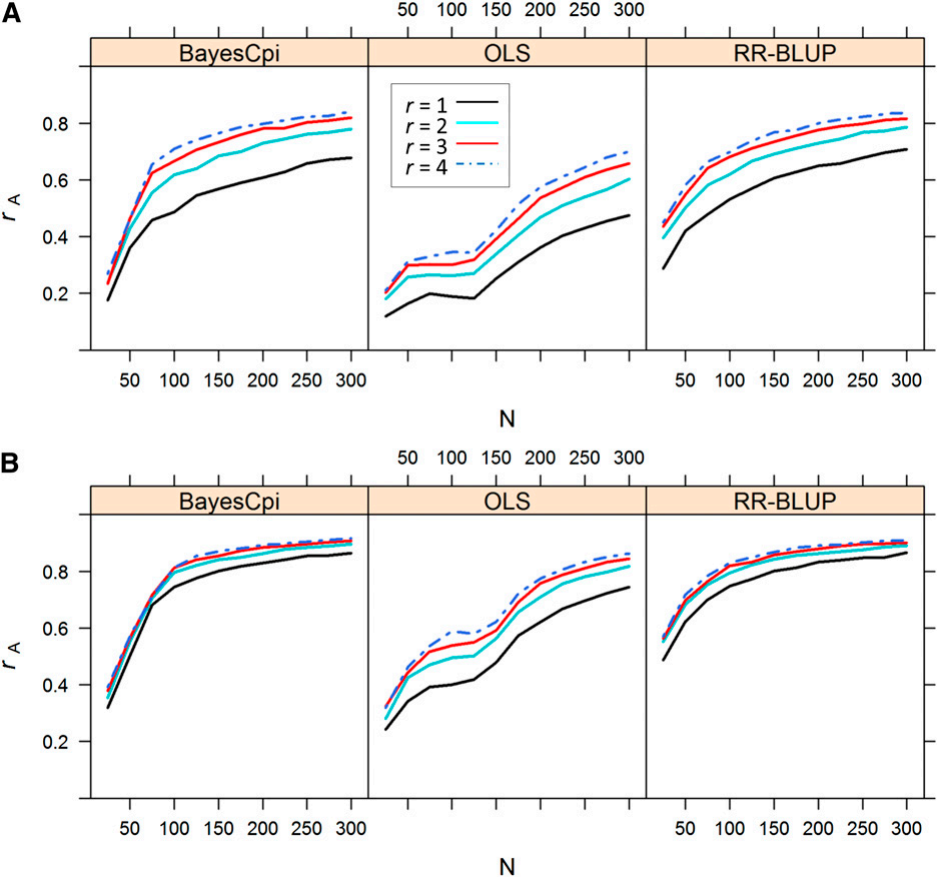
Prediction accuracy (*r*_A_) as a function of replication number and population size for each of three statistical models. (A) Heritability of single plot measurements set to 0.20. (B) Heritability of single plot measurements set to 0.60.

Genomic selection accuracies were calculated for various levels of *n* and *r* for plot heritabilities of 0.20 (Figure 1A) and 0.60 (Figure 1B). Accuracy increased with training population size and replication number as expected (Figure 1). The rate of accuracy increase in response to greater *n* varied between the different models. RR-BLUP faired quite well even when *n* was less than 75. BayesC*π*, on the other hand, performed quite poorly until *n* was greater than 75, and accuracy slowly increased beyond *n* = 100 at about the same rate as it increased for RR-BLUP. As *n* increased greater than 125, accuracy slowly increased, and the curves presented in Figure 1 show diminishing returns between *n* = 125 and *n* = 300. For the OLS model, on the other hand, accuracies remain low until *n* = 125, then continued to rapidly increase through *n* = 300.

Interestingly, a substantial boost in accuracy from *r* = 1 to *r* = 2 was observed across levels of *n* for $h_{plot}^{2}=0.20$. It was expected that the advantage in model accuracy achieved by increasing *r* = 1 to *r* = 2 would diminish as *n* increased, but the boost in accuracy was remarkably consistent. For example, increasing *r* = 1 to *r* = 2 increased *r*_A_ by 0.09 for *n* = 100:model = RR-BLUP: $h_{plot}^{2}=0.20$, by 0.09 for *n* = 200, and by 0.08 for *n* = 300. To test the extent to which increasing *r* improved *r*_A_ despite large population sizes, the simulation was taken out to very large, impractical population sizes for model = RR-BLUP: $h_{plot}^{2}=0.20$ and model = OLS: $h_{plot}^{2}=0.20$ (Figure S1). When RR-BLUP is used, there are diminishing returns in *r*_A_ by increasing *r* = 1 to *r* = 2 as *n* is increased, but only after *n* exceeds 300. Even then, *r*_A_ for *r* = 1 approaches that of *r* = 2 only very slowly, and a difference of 0.05 is still observed at *n* = 700.

One thing that is apparent in these results is that RR-BLUP achieves much greater accuracies at all levels of *n*, but the difference is much greater at relatively small values of *n*. When *n* = 100, RR-BLUP is more than 34% more accurate than the OLS model, but when *n* > 500, RR-BLUP is only 15% more accurate than the OLS model. This property of RR-BLUP indicates that resource allocation decisions will differ between traditional marker-assisted selection and genomic selection.

### Effect of tradeoff between population size and replication

In reality, breeding programs have budgets to contend with. Budgets of 250 and 500 field plots per single DH population were considered. I assumed different genotyping costs (C), expressed in field plot equivalents of 0, 0.50, and 1. A situation in which genotyping is free (*i.e.*, C = 0) could be where a population was genotyped for another reason not related to the breeding program, or a futuristic scenario where genotyping technologies have advanced to the point of *in situ* genotyping assays, for example. BayesC*π* was no longer considered for subsequent analyses because it performed so similarly to RR-BLUP, and it was decided to focus the results on one model.

**Figure 2:**
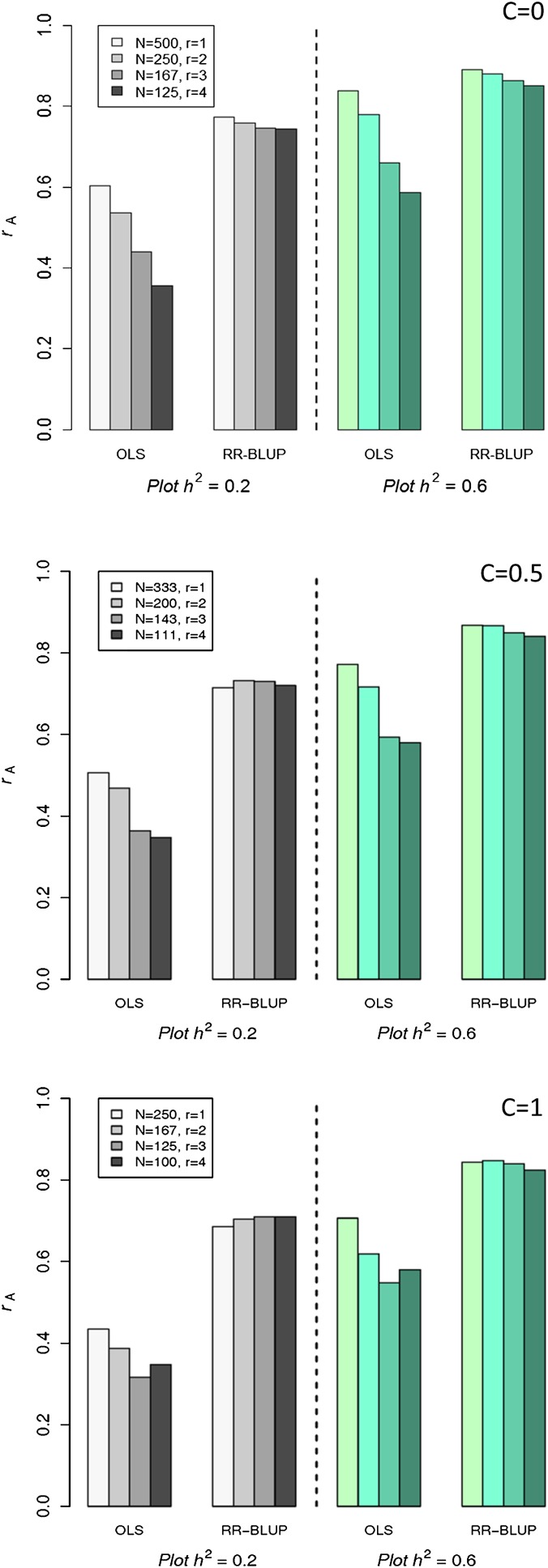
Prediction accuracy (*r*_A_) for two statistical models as affected by tradeoffs between replication (*r*) and population size (*n*) for various levels of relative genotyping costs (C) expressed in field plot equivalents. Total budget is set to 500 field plot equivalents.

When B = 500, prediction accuracy measured by cross validation was always maximized at *r* =1 and the greatest value of *n* for OLS (Figure 2). This was the case for both $h_{plot}^{2}=0.20$ and $h_{plot}^{2}=0.60$ for all levels of C. Large differences in accuracy were observed between *r* =1 and *r* =4. Prediction accuracy of RR-BLUP, on the other hand, responded very little to allocating resources between *n* and *r*. For example, when C = 0, so that there is a direct tradeoff between level of replication and population size, *n* = 500:*r* = 1 resource allocation strategy produced a model only 4% more accurate than a *n* = 125:*r* = 4 resource allocation strategy, whereas the increase in accuracy was 67% for OLS. This essentially means that slightly greater accuracy with RR-BLUP can be achieved by replicating alleles across individuals, but the advantage isn’t nearly as great as it is for OLS. Following this, whenever C > 0 so that genotyping costs reduce *n* disproportionately as *r* increases, the advantage of maximizing *n* quickly disappears. The RR-BLUP accuracies for all resource allocation strategies are remarkably similar, indicating greater flexibility in experimental design to maximize genomic selection accuracies. As can be seen in Figure 1A, rate of increase in genomic selection accuracy with increasing *n* tapers off above *n* =100, and increasing replication from *r* =1 to *r* =2 boosts accuracy by 0.09. The combination of these factors leads to almost an even tradeoff in accuracy between *n* and *r*.

The situation is only slightly different for B = 250 (Figure S2). When C = 0 and *n* can be as large as 250 so that *r* =1, a big boost in accuracy is observed for OLS. Whenever *n* is less than this for other resource allocation strategies, OLS performs quite poorly. As when B = 500, RR-BLUP is much less sensitive to tradeoffs between *n* and *r* compared to OLS. Differences in accuracy are greater, however, for B = 250. This is likely due to the fact that values of *r* greater than two force *n* to be smaller than 100, which substantially reduces accuracy (Figure 1A).

### Comparison between theoretical prediction accuracies and observed

**Figure 3:**
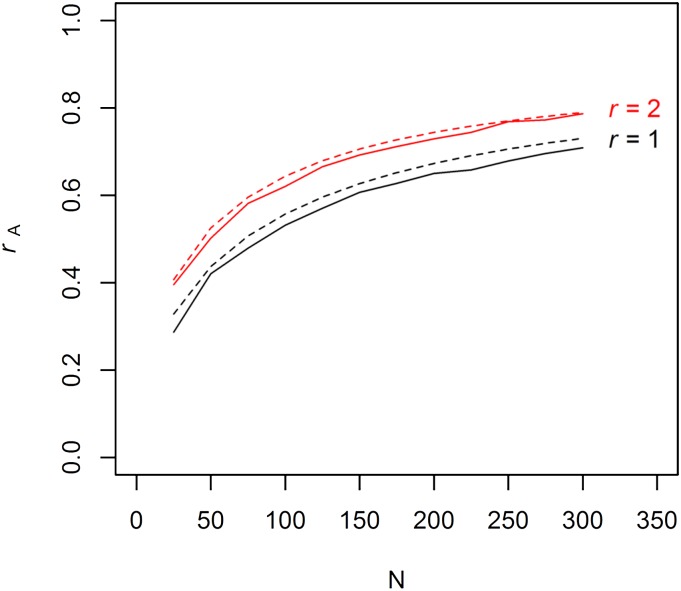
Comparison between calculated prediction accuracy (dashed lines) and observed prediation accuracy (solid lines) in simulations.

Theoretical prediction accuracies follow a very similar trajectory as that observed in the simulation (Figure 3) and are only slightly higher than the accuracy observed in the simulation, being on average 0.026 higher for *r* = 1 and 0.013 greater for *r* = 2. A key use of the theoretical accuracy calculations would be for resource allocations decisions. The *n* and *r* choices according to values of C = {0, 0.5, 1} and B = {250, 500} discussed above were used in the formula derived by Daetwyler *et al.* (2008). The resource allocation strategy (*i.e.*, *n vs. r*) predicted to give the greatest accuracy was compared with that observed in the simulation. The predicted optimal resource allocation strategy largely agreed with those observed in the simulations (Table 1). Only in three of the 12 instances was there a disagreement, and this often occurred when the difference between different levels of *n*:*r* was vanishingly small (Table 1). These results suggest that the formula derived by Daetwyler *et al.* (2008) can be used to guide genomic selection strategies in plant breeding, thereby avoiding time consuming efforts in data mining and computer simulations.

**Table 1:** Population size and number of replication numbers achieving the greatest prediction accuracy according to theoretical calculations and simulations for different resource allocation situations

	Optimal Resource Allocation Strategy (*n*:*r*)		
B – C – $h_{plot}^{2}$	Theoretical (Obs *r*_A_*^a^*)	Observed (Obs *r*_A_)	Agreement
250 – 0 – 0.20	250:1	250:1	Yes
250 – 0.5 – 0.20	100:2	100:2	Yes
250 – 1 – 0.20	83:2	83:2	Yes
250 – 0 – 0.60	250:1	250:1	Yes
250 – 0.5 – 0.60	167:1	167:1	Yes
250 – 1 – 0.60	125:1 (0.773)	83:2 (0.777)	No
500 – 0 – 0.20	500:1	500:1	Yes
500 – 0.5 – 0.20	200:2	200:2	Yes
500 – 1 – 0.20	167:2 (0.704)	125:3 (0.710)	No
500 – 0 – 0.60	500:1	500:1	Yes
500 – 0.5 – 0.60	333:1	333:1	Yes
500 – 1 – 0.60	250:1 (0.844)	167:2 (0.848)	No

Predictions in the simulations were made with the RR-BLUP model. The column titled “Agreement” indicates if the theoretical calculations agreed with what was observed in the simulation. B, total budget in field plot equivalents; C, genotyping cost in field plot equivalents; $h_{plot}^{2}$, heritability of single plot measurements; RR-BLUP, ridge regression best linear unbiased prediction.

a Prediction accuracies observed in the simulation are displayed in parenthesis for the instances in which the theoretical and observed optimal resource allocation strategies do not agree.

### Effect of varying n and r on multiple cycles of selection

**Figure 4:**
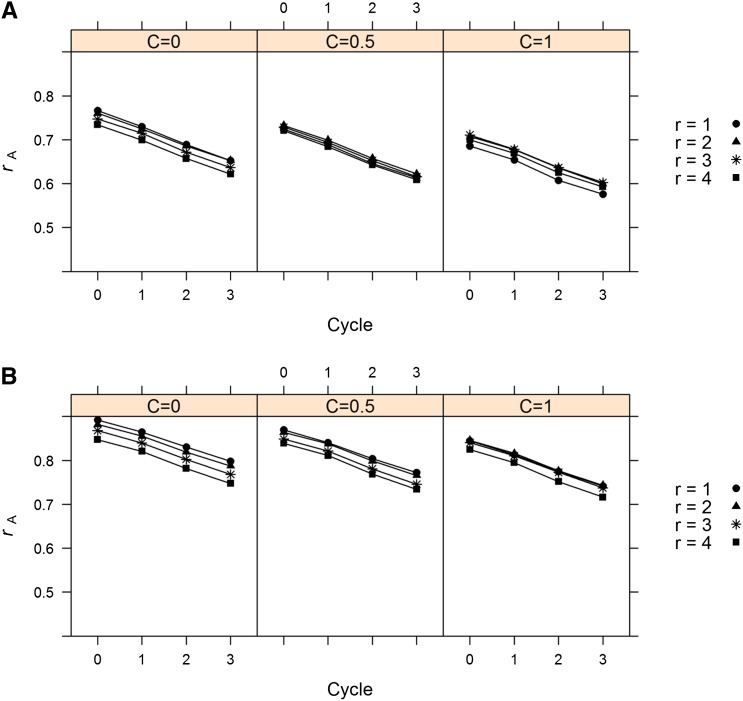
Prediction accuracy (*r*_A_) for each relative genotyping cost and resource allocation strategy across generations of random mating (Cycle). Population sizes corresponding to each level of *r* can be observed in Figure 2. Total budget was set to 500 field plot equivalents. (A) Heritability of single plot measurements set to 0.20. (B) Heritability of single plot measurements set to 0.60. Average standard error of prediction accuracies was 0.005 and ranged from 0.002 to 0.007.

Resource allocation strategies were applied to generating phenotypic data for model training. One goal of this study was to determine how different strategies, constrained by budgets, affected prediction accuracy not only in Cycle 0 but also the prediction of progenies in cycles 1 to 3. To isolate prediction accuracy from the confounding effect of shifting genetic variances caused by different selection intensities and changes in allele frequency, a model was trained in Cycle 0 followed by randomly mating the population for three generations. Models trained in Cycle 0 were applied to progenies in Cycles 1−3. No change in rank across cycles of recombination was observed for the different resource allocation strategies (Figure 4). For example, when C = 0, a *n*:*r* ratio of 500:1 was optimal across all cycles; when C = 1, a *n*:*r* ratio of 125:3 was optimal across all cycles. The exact same finding of no crossover in rank across cycles was observed for all other scenarios (Figure S3).

Genetic gain is a function of selection intensity and genetic variance in addition to selection accuracy. The relative genetic gain of two methods for performing the first cycle of selection was evaluated: selecting C0 progenies on the basis of phenotypic value alone *vs.* selection based on marker scores. The latter holds potential to be advantageous because of extensive replication at the allelic level, and therefore prediction accuracy will benefit from larger populations, whereas phenotypic selection does not. Bernardo and Yu (2007), however, reported that simulated selection based on phenotypic value alone in the first cycle was at least as successful as an index value combining phenotypic values and marker scores. In the simulations performed for this study, selection on marker scores alone in cycle 0 led to greater genetic gain than selection on phenotypic values or GEBV-phenotype indices (results not shown), and therefore all selections made in cycle 0 were based on marker scores alone.

Three options were considered for phenotyping: (1) phenotype 100% of DH lines, (2) phenotype only 75% of DH lines, (3) phenotype only 50% of DH lines. The latter two options were chosen to save resources for phenotyping, thereby allocating more resources to genotyping larger populations. Total population sizes and number of DH lines phenotyped at four levels of replication are displayed in Table 2, Table 3, Table 4, and Table 5. Surprisingly, phenotyping only a fraction of the DH population provided no benefit (Tables 2−5). This was largely because only a limited increase in selection intensity was achieved, as well as the fact that lines phenotyped and included in the training dataset were predicted with higher accuracy than lines not phenotyped.

**Table 2:** Response and prediction accuracy of several resource allocation and phenotyping strategies for a budget of 250 total plot units, relative genotyping cost of 0.50, and plot heritability of 0.20 (*i.e.*, B = 250, C = 0.50, $h_{plot}^{2}$=0.20)

%Pheno.*^a^*	*r*	*n*	*n*_Ph_*^b^*	*i*	Prediction Accuracy of Phenotyped DHs*^c^*	Prediction Accuracy of Nonphenotyped DHs*^d^*	Cycle 1 Mean	Cycle 2 Mean	Cycle 3 Mean	Standard Error of Cycle 3 Mean
50	1	250	125	2.15	0.66	0.58	1.28	1.66	1.90	0.021
50	2	167	84	1.99	0.70	0.59	1.22	1.63	1.92	0.020
50	3	125	62	1.86	0.74	0.59	1.20	1.64	1.92	0.019
50	4	100	50	1.75	0.76	0.58	1.13	1.56	1.89	0.020
75	1	200	150	2.06	0.67	0.60	1.32	1.73	2.02	0.020
75	2	125	94	1.86	0.72	0.61	1.26	1.70	2.00	0.018
75	3	91	68	1.71	0.75	0.61	1.21	1.67	1.96	0.019
75	4	71	53	1.59	0.77	0.58	1.13	1.60	1.90	0.020
100	1	167	167	1.99	0.68	0.62	1.33	1.76	2.03*	0.019
100	2	100	100	1.75	0.72	0.63	1.26	1.71	2.02	0.019
100	3	71	71	1.59	0.75	0.62	1.17	1.65	1.97	0.019
100	4	56	56	1.46	0.77	0.61	1.12	1.59	1.90	0.019

Predictions were made with RR-BLUP. Resource allocation strategy with highest cycle 3 mean is indicated by asterisk (*) in Cycle 3 mean column. All resource allocation strategies producing genetic gain not significantly different from the greatest genetic gain observed are underlined. Units are in C0 genetic SDs. RR-BLUP, ridge regression best linear unbiased prediction; GEBVs, genomic estimated breeding value.

a Percentage of DH population phenotyped. DH lines not phenotyped were genotyped and genomic selection model was applied to calculate their GEBVs.

b Number of DH lines phenotyped (*i.e.*, %Pheno × *n* rounded to nearest whole integer).

c Prediction accuracy of DH lines that were phenotyped and included in the model training dataset.

d Prediction accuracy of DH lines that were not phenotyped.

**Table 3:** Response and prediction accuracy of several resource allocation and phenotyping strategies for a budget of 250 total plot units, relative genotyping cost of 1, and plot heritability of 0.20 (*i.e.*, B = 250, C = 1, $h_{plot}^{2}$= 0.20)

%Pheno.*^a^*	*r*	*n*	*n*_Ph_*^b^*	*i*	Prediction Accuracy of Phenotyped DHs*^c^*	Prediction Accuracy of Nonphenotyped DHs*^d^*	Cycle 1 Mean	Cycle 2 Mean	Cycle 3 Mean	Standard Error of Cycle 3 Mean
50	1	167	84	1.99	0.61	0.51	1.05	1.44	1.64	0.021
50	2	125	62	1.86	0.68	0.54	1.11	1.52	1.76	0.021
50	3	100	50	1.75	0.73	0.56	1.09	1.53	1.78	0.020
50	4	83	42	1.67	0.75	0.55	1.08	1.51	1.78	0.020
75	1	143	107	1.92	0.62	0.54	1.11	1.52	1.73	0.020
75	2	100	75	1.75	0.70	0.58	1.16	1.60	1.86	0.019
75	3	77	58	1.63	0.73	0.57	1.10	1.55	1.81	0.019
75	4	62	46	1.52	0.76	0.57	1.06	1.53	1.80	0.019
100	1	125	125	1.86	0.64	0.56	1.16	1.59	1.81	0.020
100	2	83	83	1.67	0.71	0.60	1.15	1.61	1.90	0.019
100	3	62	62	1.52	0.75	0.60	1.14	1.62	1.94*	0.018
100	4	50	50	1.40	0.77	0.59	1.04	1.52	1.84	0.019

Predictions were made with RR-BLUP. Resource allocation strategy with highest cycle 3 mean is indicated by asterisk (*) in Cycle 3 mean column. All resource allocation strategies producing genetic gain not significantly different from the greatest genetic gain observed are underlined. Units are in C0 genetic SDs. RR-BLUP, ridge regression best linear unbiased prediction; GEBVs, genomic estimated breeding value.

a Percentage of DH population phenotyped. DH lines not phenotyped were genotyped and genomic selection model was applied to calculate their GEBVs.

b Number of DH lines phenotyped (*i.e.*, %Pheno × *n* rounded to nearest whole integer).

c Prediction accuracy of DH lines that were phenotyped and included in the model training dataset.

d Prediction accuracy of DH lines that were not phenotyped.

**Table 4:** Response and prediction accuracy of several resource allocation and phenotyping strategies for a budget of 500 total plot units, relative genotyping cost of 0.50, and plot heritability of 0.20 (*i.e.*, B = 500, C = 0.50, $h_{plot}^{2}$=0.20)

%Pheno.*^a^*	*r*	*n*	*n*_Ph_*^b^*	*i*	Prediction Accuracy of Phenotyped DHs*^c^*	Prediction Accuracy of Nonphenotyped DHs*^d^*	Cycle 1 Mean	Cycle 2 Mean	Cycle 3 Mean	Standard Error of Cycle 3 Mean
50	1	500	250	2.42	0.72	0.68	1.61	1.99	2.33	0.019
50	2	333	166	2.27	0.77	0.70	1.62	2.03	2.39	0.018
50	3	250	125	2.15	0.80	0.71	1.58	2.01	2.39	0.018
50	4	200	100	2.06	0.81	0.70	1.51	1.94	2.32	0.020
75	1	400	300	2.34	0.74	0.71	1.63	2.04	2.39	0.018
75	2	250	188	2.15	0.78	0.73	1.57	2.01	2.39	0.017
75	3	182	136	2.02	0.80	0.72	1.52	1.97	2.36	0.018
75	4	143	107	1.92	0.82	0.72	1.50	1.96	2.36	0.018
100	1	333	333	2.27	0.76	0.72	1.65	2.08	2.45*	0.019
100	2	200	200	2.06	0.79	0.73	1.59	2.04	2.44	0.017
100	3	143	143	1.92	0.81	0.73	1.50	1.98	2.38	0.017
100	4	111	111	1.80	0.82	0.72	1.45	1.92	2.33	0.017

Predictions were made with RR-BLUP. Resource allocation strategy with highest cycle 3 mean is indicated by asterisk (*) in Cycle 3 mean column. All resource allocation strategies producing genetic gain not significantly different from the greatest genetic gain observed are underlined. Units are in C0 genetic SDs. RR-BLUP, ridge regression best linear unbiased prediction; GEBVs, genomic estimated breeding value.

a Percentage of DH population phenotyped. DH lines not phenotyped were genotyped and genomic selection model was applied to calculate their GEBVs.

b Number of DH lines phenotyped (*i.e.*, %Pheno × *n* rounded to nearest whole integer).

c Prediction accuracy of DH lines that were phenotyped and included in the model training dataset.

d Prediction accuracy of DH lines that were not phenotyped.

**Table 5:** Response and prediction accuracy of several resource allocation and phenotyping strategies for a budget of 500 total plot units, relative genotyping cost of 1, and plot heritability of 0.20 (*i.e.*, B = 500, C = 1, $h_{plot}^{2}$=0.20)

%Pheno.*^a^*	*r*	*n*	*n*_Ph_*^b^*	*i*	Prediction Accuracy of Phenotyped DHs*^c^*	Prediction Accuracy of Nonphenotyped DHs*^d^*	Cycle 1 Mean	Cycle 2 Mean	Cycle 3 Mean	Standard Error of Cycle 3 Mean
50	1	333	166	2.27	0.68	0.61	1.42	1.80	2.09	0.021
50	2	250	125	2.15	0.75	0.66	1.46	1.88	2.21	0.019
50	3	200	100	2.06	0.78	0.68	1.44	1.88	2.21	0.019
50	4	167	84	1.99	0.80	0.68	1.44	1.87	2.25	0.018
75	1	286	214	2.21	0.71	0.66	1.48	1.89	2.20	0.021
75	2	200	150	2.06	0.76	0.69	1.49	1.93	2.29	0.019
75	3	154	116	1.95	0.79	0.70	1.46	1.91	2.28	0.017
75	4	125	94	1.86	0.81	0.69	1.41	1.87	2.26	0.017
100	1	250	250	2.15	0.73	0.68	1.53	1.96	2.29	0.018
100	2	167	167	1.99	0.77	0.71	1.49	1.95	2.31*	0.018
100	3	125	125	1.86	0.80	0.71	1.42	1.90	2.28	0.018
100	4	100	100	1.75	0.81	0.71	1.40	1.88	2.28	0.018

Predictions were made with RR-BLUP. Resource allocation strategy with highest cycle 3 mean is indicated by asterisk in Cycle 3 mean column. All resource allocation strategies producing genetic gain not significantly different from the greatest genetic gain observed are underlined. Units are in C0 genetic SDs. RR-BLUP, ridge regression best linear unbiased prediction; GEBVs, genomic estimated breeding value.

a Percentage of DH population phenotyped. DH lines not phenotyped were genotyped and genomic selection model was applied to calculate their GEBVs.

b Number of DH lines phenotyped (*i.e.*, %Pheno × *n* rounded to nearest whole integer).

c Prediction accuracy of DH lines that were phenotyped and included in the model training dataset.

d Prediction accuracy of DH lines that were not phenotyped.

With respect to comparing genetic gain across resource allocation strategies, results similar to those for prediction accuracy were found. That is, a degree of flexibility exists with equivalent genetic gains being observed over a number of different strategies. For example, when B = 250 and C = 0.50, no difference was observed between *n* = 167:*r* = 1 and *n* = 100:*r* = 2 (Table 2). The same was observed for B = 500 and both levels of C. When B = 250 and C = 1, however, the greatest genetic gain was observed for *n* = 62 and *r* = 3. The small population size here was offset by the increase in accuracy through additional replication. In summary, if genotyping is relatively inexpensive (*i.e.*, half the cost of phenotyping a single experimental unit), one or two replications can be used without any difference in genetic gain. Allocating more resources to additional replication is not advisable. If genotyping costs at least as much as phenotyping one experimental unit, replication is beneficial, especially when budgets are small. If the budget is large, several different strategies can be used without sacrificing total genetic gain. It appears increases in selection intensity through larger population sizes are offset by increases in prediction accuracy through more precise phenotyping.

## Discussion

Genomic selection can be incorporated into a breeding program in several different ways including, but not limited to, predicting and selecting within biparental populations (Bernardo and Yu 2007; Heffner *et al.* 2011), across multiple related biparental populations (Albrecht *et al.* 2011) and among a panel of inbred lines or accessions (Riedelsheimer *et al.* 2012). When genomic selection is applied to any of these populations, GEBVs can be used to select progenies genotyped but not phenotyped, or GEBVs can augment or replace phenotypes if they are more predictive of true breeding values. The latter may be advantageous if large populations and a high degree of allelic replication provide highly accurate allelic effect estimates (Gimelfarb and Lande 1994). In either case, a key question is whether resources should be used to maximize population size or if some resources should be allocated to replication in order to increase heritability.

This simulation study on a single biparental population of DH lines showed that when the objective is to predict nonphenotyped progenies, plot-based heritability is low, and cost of genotyping is at least 50% that of phenotyping an experimental unit, RR-BLUP prediction accuracy slightly benefits from replication. When genotyping is free or $h_{plot}^{2}$ is high, allocating resources to a larger population maximizes prediction accuracy. The differences between resource allocation strategies, however, were quite small across scenarios. This stands in contrast to what has been previously reported for QTL detection power (Knapp and Bridges 1990; Schon *et al.* 2004), as well as what was found for a conventional marker-assisted selection approach tested in the present study. When marker-based selection was carried out by using OLS estimation and a model selection algorithm, prediction accuracy was clearly and consistently maximized when all resources were allocated to maximizing *n* and minimizing *r*. Figure 1 shows that accuracy of OLS and RR-BLUP responded to replication approximately equally. A large difference between the two models, especially when $h_{plot}^{2}$= 0.20, is that OLS accuracy didn’t respond until *n* exceeded 125, whereas RR-BLUP accuracy increased between *n* = 25 and *n* = 125, and actually began to level off just above *n* = 125. Accuracy of OLS continued to steadily increase until *n* was greater than 300. Because *n* was always less than or equal to 250 when *r* =2, boosting *n* by decreasing *r* provided a substantial benefit in accuracy to OLS, whereas RR-BLUP benefited less at these larger population sizes.

It is well known that OLS estimates of marker effects and model selection algorithms suffer from inability to explain sufficient proportions of genetic variance for complex traits. This is primarily caused by lack of statistical power to detect small-effect QTL comprising genetic variation of complex, agronomically important traits. Power is especially low when population size is relatively small. In fact, Utz *et al.* (2000) found that power was insufficient for detecting grain yield QTL even at population sizes of 300. Compounding the problem of insufficient power is upward bias of QTL effect estimation, which is inversely related to power (Beavis 1994; Melchinger *et al.* 1998). As population size decreases, both QTL detection power diminishes and estimation bias increases, resulting in marker-assisted selection model of dramatically reduced prediction accuracy. An RR-BLUP genomic selection model, on the other hand, does not suffer from lack of statistical power because all marker effects are estimated and used for prediction. Population size does affect marker effect estimation accuracy and thus genomic prediction accuracy (Lorenz *et al.* 2012; Lorenzana and Bernardo 2009; Heffner *et al.* 2011), but apparently not as much as power and estimation accuracy of OLS MAS models.

A lack of rank changes among resource allocation strategies across generations of random mating was unexpected. It was expected that a resource allocation strategy better able to separate markers in terms of their effects and thus give greater weight to markers more closely linked to QTL would perform better on progenies several generations of random mating removed from the training population. It was hypothesized that maximizing population size, thus creating more recombination events for increased resolution, would always produce a model with superior prediction accuracies on progenies several generations removed from the training population. This was observed by Zhong *et al.* (2009), where more resources allocated to replication improved prediction when the training and validation populations were only one generation removed, but allocating more resources to larger population size resulted in greater prediction accuracy when training and validation populations were separated by four generations of random mating. These authors used a different population structure (several related biparental populations derived from actual barley genotypes) and did not account for genotyping cost. Another example of rank changes across generations of random mating is included in Habier *et al.* (2007), where different genomic selection models performed similarly when training and validation populations were removed by one or less generations, but BayesB performed markedly better as the number of generations between training and validation populations increased. This was because BayesB put more weight on markers more closely linked to QTL. Despite these theoretical expectations, I observed no change in rank across generations, indicating that a particular resource allocation strategy will be superior across at least three generations. One explanation is the high level of linkage disequilibrium (LD) between markers and QTL and an insufficient number of generations to break up linkages, although predictions accuracies generally declined as expected. The number of effective factors was 28, but the number of simulated QTL was 100, resulting in a situation where QTL are expected to be in high LD with several markers.

With respect to total genetic gain, simulations under the budgets and genotyping:phenotyping cost ratios considered in this study again indicated a degree of flexibility in terms of resource allocation. In three of the four cases studied (Tables 2−5), multiple strategies were not statistically different from one another in terms of genetic gain. In each of these three cases, maximizing population size and phenotyping 100% of the DH lines resulted in at least as much genetic gain as any other strategy. When the budget was relatively small and genotyping cost was relatively high, it was beneficial to conduct three replications to increase heritability. Phenotyping less than 100% of the DH lines that were genotyped to save resources provided little benefit. It appears this is because prediction accuracy of lines included in the training dataset was always substantially higher than those not included. To my knowledge, in no other studies have authors specifically investigated this in the context of either genomic selection or marker-assisted selection, so an attempt at an intuitive explanation is warranted. Prediction accuracy is partly a function of LD between markers and QTL. Linkage disequilibrium between markers and QTL not physically linked can be generated by random genetic drift, or sampling. Because we are dealing with training populations of finite size, the sampling effect can generate slight LD between unlinked markers and QTL. This source of LD is useful for purposes of prediction within the same sample of individuals that generated that spurious LD. However, when marker effects are applied to individuals outside the original sample of individuals, the same exact spurious pattern of LD between unlinked markers and QTL no longer exists. Rather, this source of LD just generates noise, reducing marker effect estimation accuracy Increasing the heritability of measurements increases the accuracy derived from this source of LD when predictions are applied within the training population. However, when predictions are applied outside the original sample, increasing population size has a larger effect because it reduces noise caused by spurious LD (J-L. Jannink, personal communication).

It is acknowledged that one expense not considered for allocating resources was cost of developing DH lines, which is certainly a considerable cost for breeding programs. One reason this expense was not considered is because of uncertainty in its relative cost and greatly varying costs between organizations. Nevertheless, the finding of little benefit to phenotyping only a subset of the genotyped population would not change, and actually factoring in cost of DH line development would only make phenotyping the entire population more beneficial.

An important factor not considered in this study, and all studies on resource allocation for MAS, is genotype-by-environment interaction. The simulations carried out implicitly assume that the phenotypic data comprising the training dataset was collected in an environment perfectly representative of the target environment. In an actual breeding program, multiple locations and possibly multiple years are needed so allelic effects are estimated across a set of environments representative of those in which the cultivar is grown in subsequent seasons. Results reported herein pertain to the effect of replication within any given environment. The effect of genotype-by-environment interaction on the allocation of resources across environments needs further study using data from actual field trials. For example, is it more efficient to evaluate 100 different progenies in each of five environments for a total population size of 500, or evaluate 100 common progenies at five environments? The former strategy allows for much larger population sizes, but each progeny is only evaluated in one environment. Given that alleles are shared across progenies, the strategy of spreading populations across environments may be more effective.

The main finding of this simulation study applicable to plant breeding programs is the flexibility found in resource allocation in genomic selection for highly polygenic traits. Unlike with traditional MAS approaches, where clearly population size should be maximized, genomic prediction accuracy and total genetic gain are less affected by allocating more resources to replication for low heritability traits like grain yield, and in some cases increased replication results in the maximum genetic gain. It was also shown that the deterministic calculations for prediction accuracy (Daetwyler *et al.* 2008) are useful for comparing resource allocation strategies in biparental populations.

## Supplementary Material

Supporting Information
