# Supplementary material for: Resource Allocation for Maximizing Prediction Accuracy and Genetic Gain of Genomic Selection in Plant Breeding: A Simulation Experiment
Source: G3 (Bethesda). 2013 Mar 1;3(3):481–91. doi: 10.1534/g3.112.004911 (PMC3583455; doi:10.1534/g3.112.004911)
Supplement: Supporting Information [file supp_3_3_481__index.html]

Supporting Information 

# Resource Allocation for Maximizing Prediction Accuracy and Genetic Gain of Genomic Selection in Plant Breeding: A Simulation Experiment

## Supporting Information for Lorenz, 2013

**Files in this Data Supplement:**

- Supporting Information - Figures S1-S3 and Files S1-S2 (PDF, 289 KB)
- Figure S1 - Prediction accuracy (*r*A) as a function of replication number and population size for each of two statistical models (PDF, 97 KB)
- Figure S2 - Prediction accuracy (*r*A) for two statistical models as affected by tradeoffs between replication (*r*) and population size (*n*) for various levels of relative genotyping costs (C) expressed in field plot equivalents (PDF, 136 KB)
- Figure S3 - Prediction accuracy (*r*A) for each relative genotyping cost and resource allocation strategy across generations of random mating (Cycle) (PDF, 150 KB)
- File S1 - R script containing functions used in the simulation (.r, 22 KB)
- File S2 - User script executing the functions (.r, 6 KB)
