# Supplementary material for: Resource Allocation for Maximizing Prediction Accuracy and Genetic Gain of Genomic Selection in Plant Breeding: A Simulation Experiment
Source: G3 (Bethesda). 2013 Mar 1;3(3):481–91. doi: 10.1534/g3.112.004911 (PMC3583455; doi:10.1534/g3.112.004911)
Supplement: Supporting Information [file supp_3.3.481_004911SI.pdf]

G3·Genes | Genomes | Genetics

Resource allocation for maximizing prediction accuracy and genetic gain of genomic selection in plant breeding: A simulation experiment

Aaron J. Lorenz

Department of Agronomy and Horticulture, University of Nebraska, Lincoln, NE 68583

DOI: **10.1534/g3.112.004911**

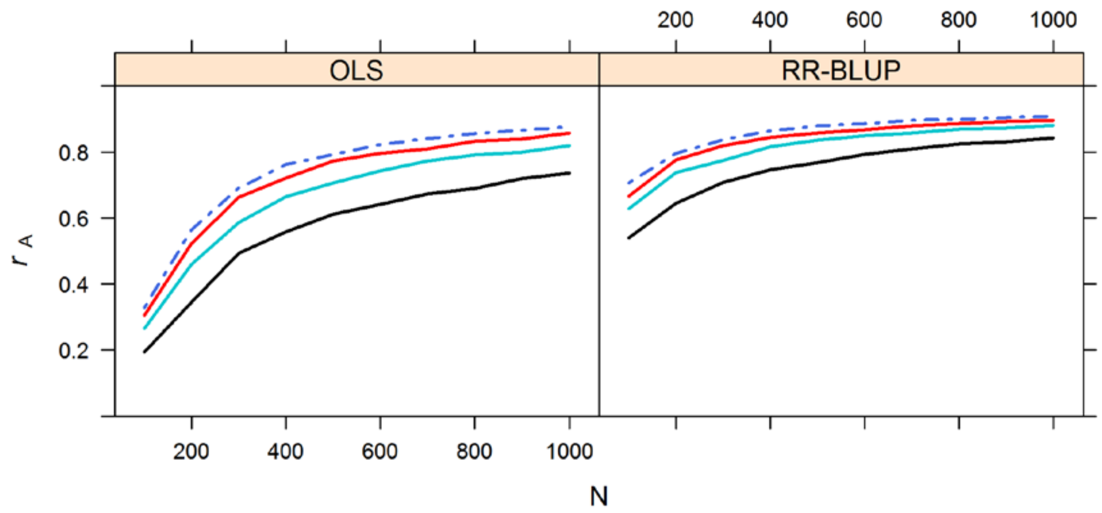

Figure S1 Prediction accuracy (r_A) as a function of replication number and population size for each of two statistical models. Black = 1 rep, Teal = 2 reps, Red = 3 reps, Dashed blue = 4 reps.

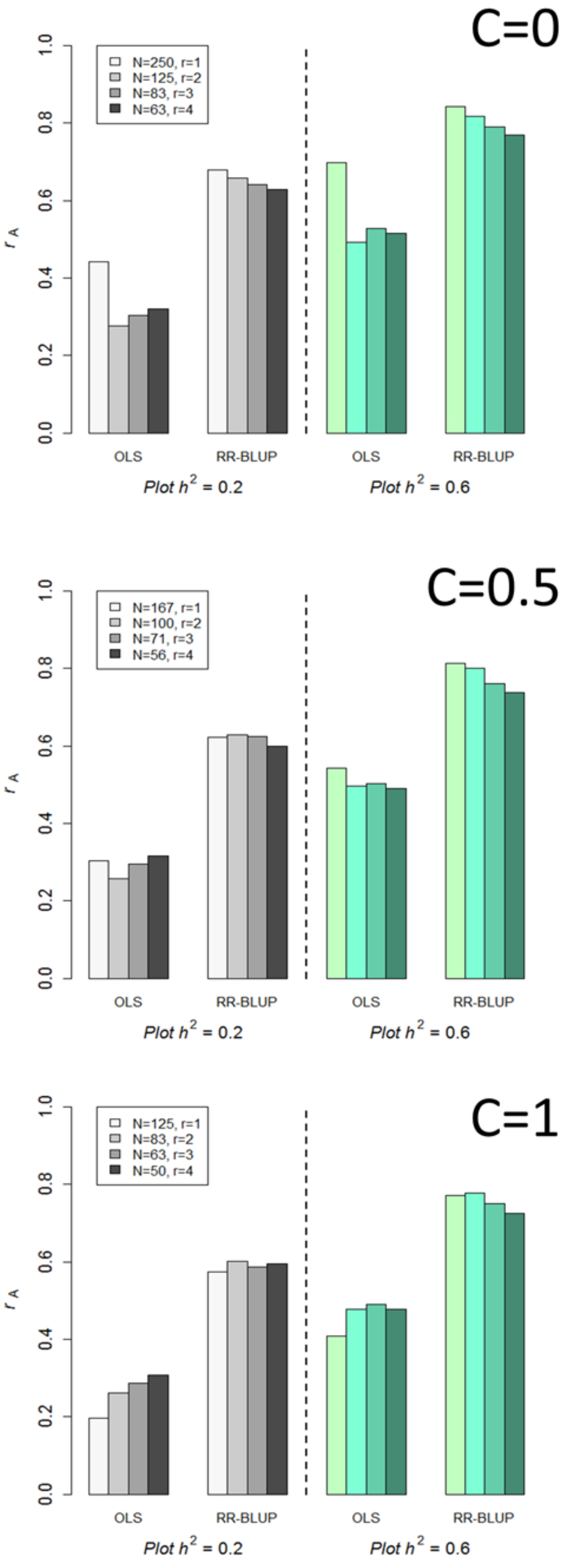

Figure S2 Prediction accuracy (r_A) for two statistical models as affected by tradeoffs between replication (*r*) and population size (*n*) for various levels of relative genotyping costs (C) expressed in field plot equivalents. Total budget is set to 250 field plot equivalents.

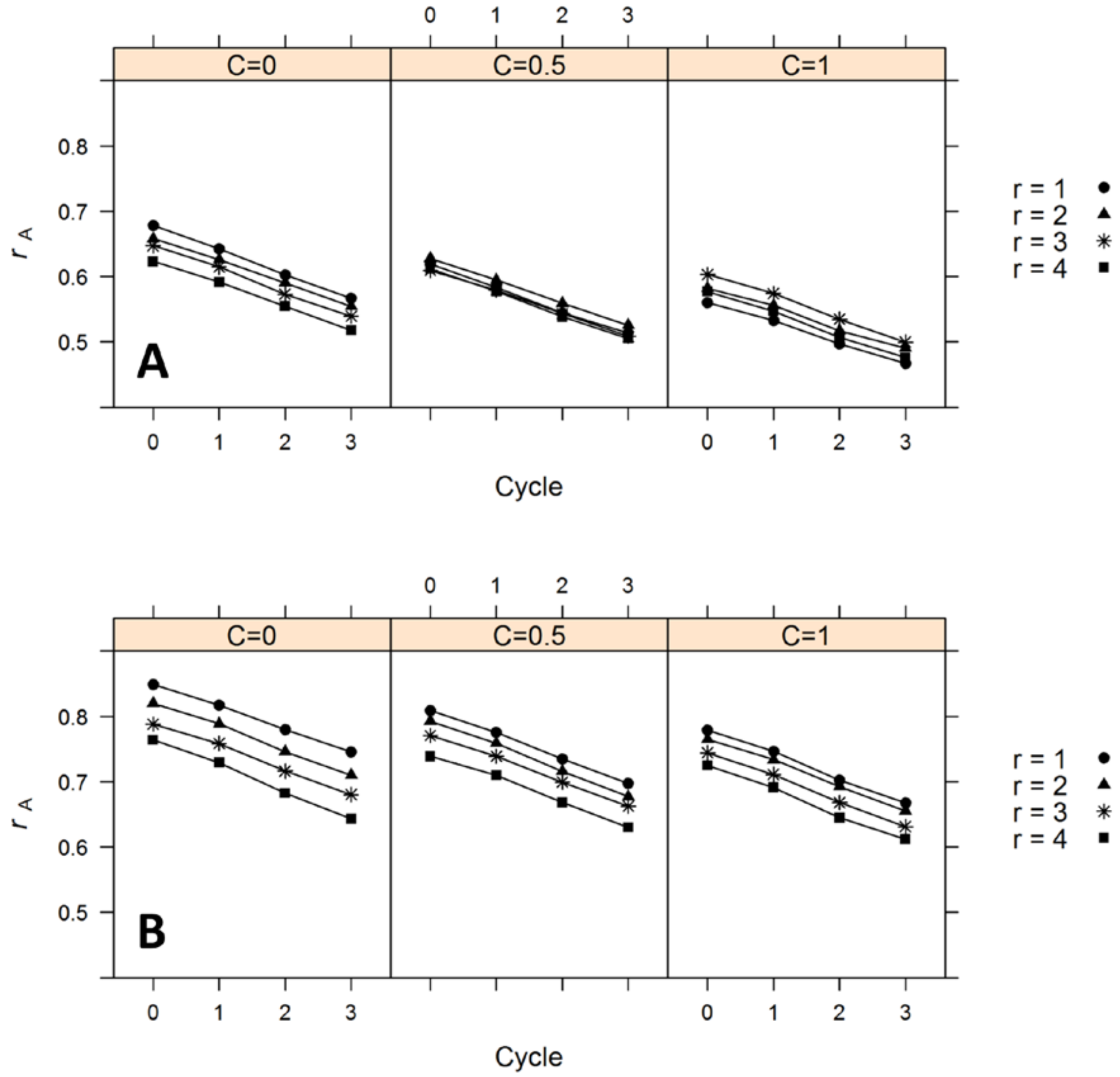

Figure S3 Prediction accuracy (r_A) for each relative genotyping cost and resource allocation strategy across generations of random mating (Cycle). Population sizes corresponding to each level of *r* can be observed in Figure 2. Total budget was set to 250 field plot equivalents. Panel A: Heritability of single plot measurements set to 0.20. Panel B: Heritability of single plot measurements set to 0.60. Average standard error of prediction accuracies was 0.006 and ranged from 0.003 to 0.008.

Files S1-S2

File S1: R script containing functions used in the simulation

File S2: User script executing the functions

Files S1-S2 are available for download at http://www.g3journal.org/lookup/suppl/doi:10.1534/g3.112.004911/-/DC1.
