# Supplementary material for: Resource Allocation for Maximizing Prediction Accuracy and Genetic Gain of Genomic Selection in Plant Breeding: A Simulation Experiment
Source: G3 (Bethesda). 2013 Mar 1;3(3):481–91. doi: 10.1534/g3.112.004911 (PMC3583455; doi:10.1534/g3.112.004911)
Supplement: Supporting Information [file supp_3.3.481_FigureS1.pdf]

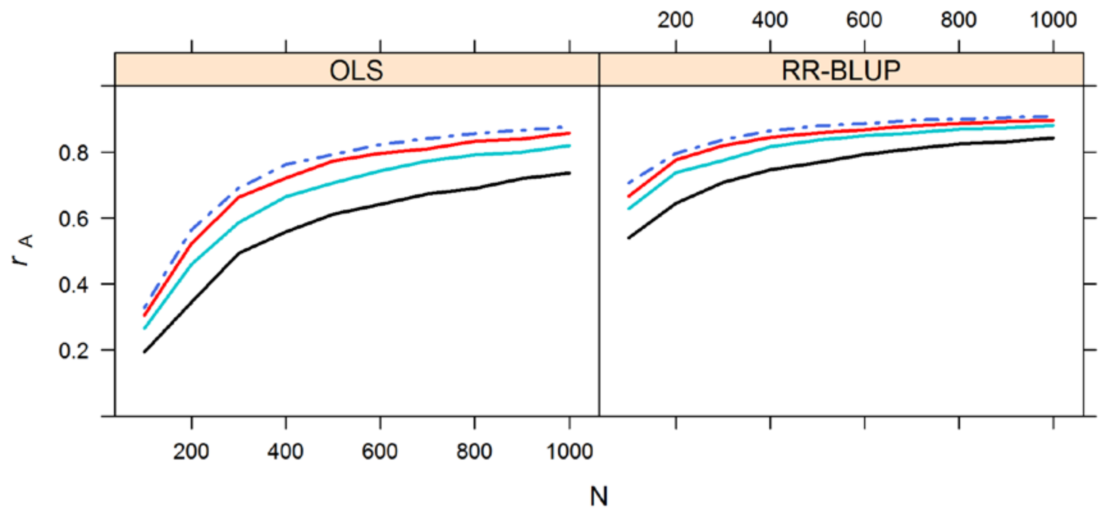

Figure S1 Prediction accuracy (r_A) as a function of replication number and population size for each of two statistical models. Black = 1 rep, Teal = 2 reps, Red = 3 reps, Dashed blue = 4 reps.
