# Supplementary material for: Resource Allocation for Maximizing Prediction Accuracy and Genetic Gain of Genomic Selection in Plant Breeding: A Simulation Experiment
Source: G3 (Bethesda). 2013 Mar 1;3(3):481–91. doi: 10.1534/g3.112.004911 (PMC3583455; doi:10.1534/g3.112.004911)
Supplement: Supporting Information [file supp_3.3.481_FigureS2.pdf]

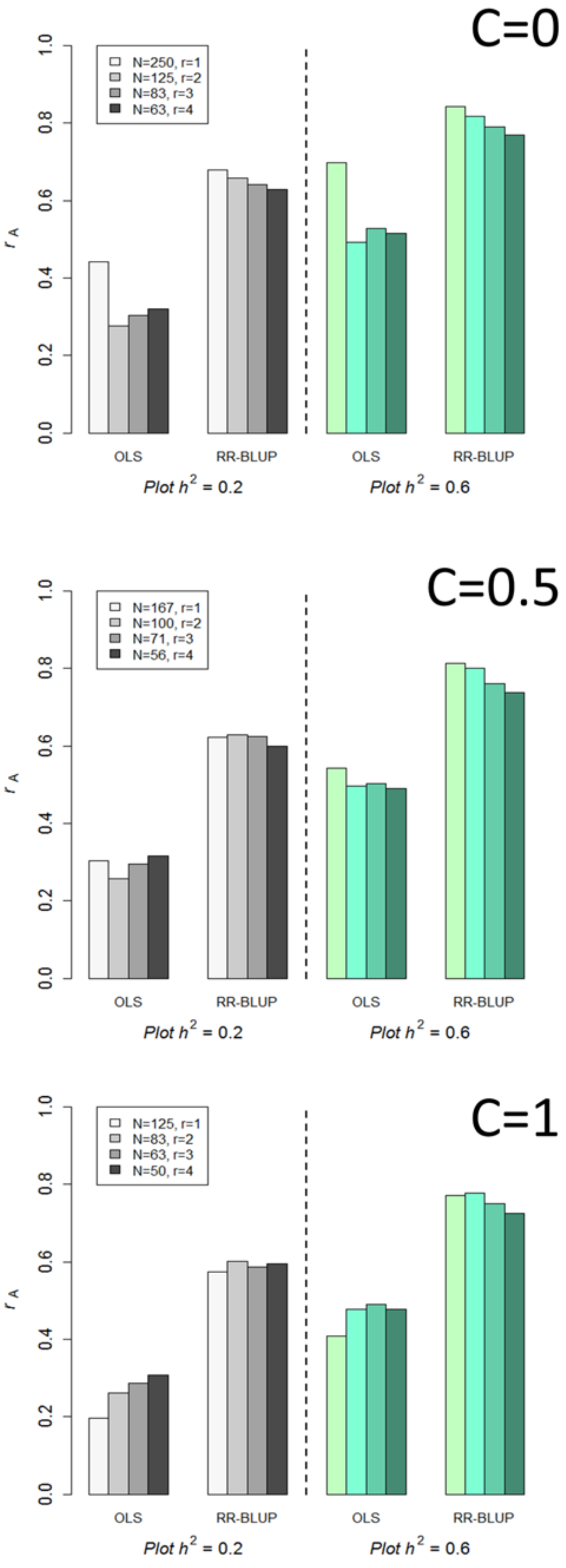

Figure S2 Prediction accuracy (r_A) for two statistical models as affected by tradeoffs between replication (*r*) and population size (*n*) for various levels of relative genotyping costs (C) expressed in field plot equivalents. Total budget is set to 250 field plot equivalents.
