# Supplementary material for: Resource Allocation for Maximizing Prediction Accuracy and Genetic Gain of Genomic Selection in Plant Breeding: A Simulation Experiment
Source: G3 (Bethesda). 2013 Mar 1;3(3):481–91. doi: 10.1534/g3.112.004911 (PMC3583455; doi:10.1534/g3.112.004911)
Supplement: Supporting Information [file supp_3.3.481_FigureS3.pdf]

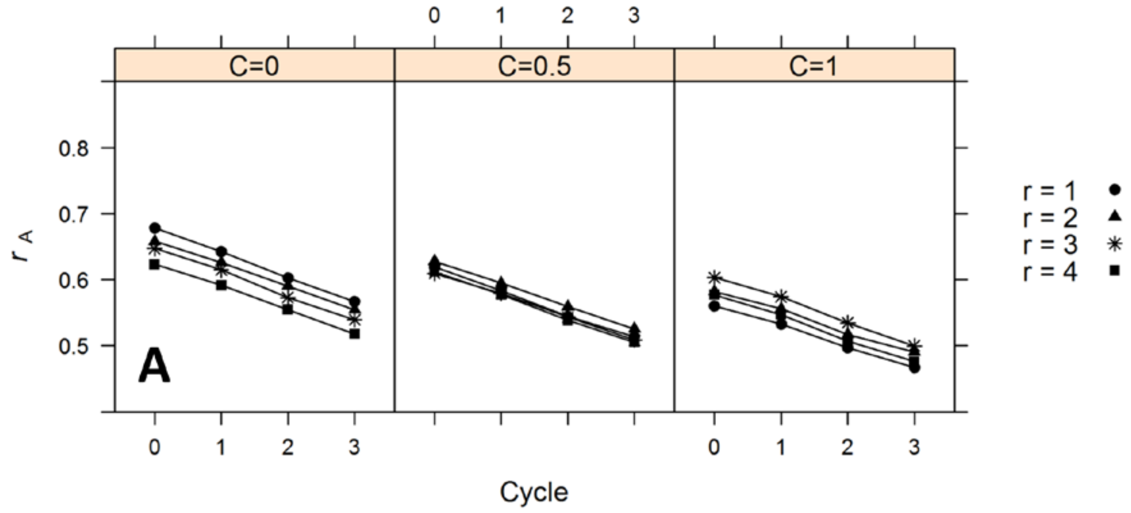

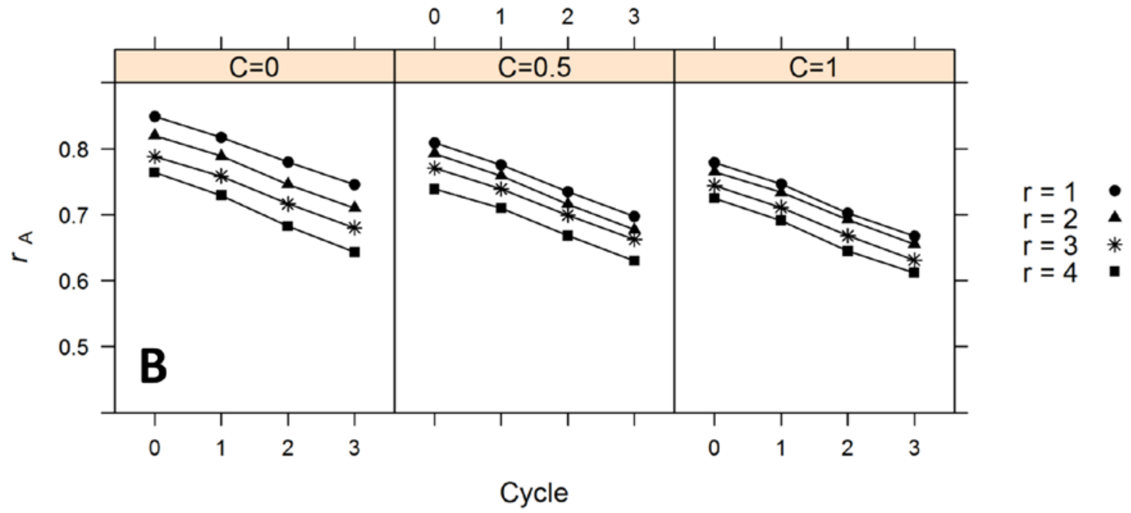

Figure S3 Prediction accuracy (r_A) for each relative genotyping cost and resource allocation strategy across generations of random mating (Cycle). Population sizes corresponding to each level of *r* can be observed in Figure 2. Total budget was set to 250 field plot equivalents. Panel A: Heritability of single plot measurements set to 0.20. Panel B: Heritability of single plot measurements set to 0.60. Average standard error of prediction accuracies was 0.006 and ranged from 0.003 to 0.008.
